# Evaluation of the MMI Symani^®^ robotic microsurgical system for coronary-bypass anastomoses in a cadaveric porcine model

**DOI:** 10.1007/s11701-024-01921-x

**Published:** 2024-04-10

**Authors:** Melanie Rusch, Grischa Hoffmann, Henning Wieker, Matthias Bürger, Sebastian Kapahnke, Rouven Berndt, René Rusch

**Affiliations:** 1 Clinic of Vascular and Endovascular Surgery, University Hospital Schleswig-Holstein, Campus Kiel, Arnold-Heller-Str 3, Hs C, D-24105 Kiel, Germany; 2 Clinic of Cranio-Maxillo-Facial Surgery, University Hospital Schleswig-Holstein, Campus Kiel, Arnold-Heller-Strasse 3, 24105 Kiel, Germany; 3 Kurt-Semm-Center for Laparoscopic and Robotic-Assisted Surgery, University Hospital Schleswig-Holstein, Campus Kiel, Arnold-Heller-Str 3, 24105 Kiel, Germany

**Keywords:** Minimal invasive microsurgery, Coronary surgery, Microanastomoses, Robotic surgery, Vascular surgery

## Abstract

The MMI Symani^®^ is a recently approved robotic microsurgical system for surgical procedures in adults. The system enables the surgeon to create microanastomoses. Clinical applications so far include lymphatic vessels surgery and the creation of special flap plastics. The use of the system in coronary arteries has not yet been assessed. The aim of this preclinical study was to evaluate the applicability of the Symani^®^ surgical system in the creation of coronary anastomoses a cadaveric porcine model. A total of 12 anastomoses were performed by three senior cardiovascular surgeons on the left main coronary artery of three porcine hearts. Artificial bypasses (diameter 1 mm) were performed to the left main trunk. The anastomoses were performed with the Symani^®^ surgical system. Evaluation included procedure times and anastomosis leakage. All anastomoses could be successfully performed. The procedure time decreased due to the learning curve between the first anastomosis 47:28 ± 5:30 min and the last anastomosis 22:37 ± 3:25 min. The final evaluation of the anastomoses showed excellent results with low leakage. The quality of the anastomosis also improved in relation to the increasing learning curve. The Symani^®^ surgical system could be used to create coronary anastomoses in an acceptable time frame and without technical failures. Hence, the system appears feasible for conventional coronary surgery. Further studies in animal models are mandatory prior to clinical application.

## Introduction

Robotic-assisted surgery has been increasingly established in many surgical disciplines and represents an important therapeutic approach [1]. However, this technique has not been fully established in cardiovascular surgery and is currently significantly underrepresented [2]. In this context, minimally invasive coronary surgery offers a surgical approach to minimize access trauma and to reduce both postoperative pain and hospital stay [3]. Nowadays, various clinical concepts have been developed with a multitude of incision approaches, cannulation options and respectively beating procedures, which allow complex robotic surgery in the cardiovascular field [4]. Except for a few highly specialized centers, the use of robotic-assisted systems in coronary surgery doesn’t play a significant role, although it has been evaluated for the past twenty years [2]. Currently, available robotic systems are reaching the limits of feasibility for coronary anastomoses [5, 6].

Generally, robotic systems offer attractive features for coronary surgery in terms of surgical precision, magnified 3D vision and downscaling of movements that are now implied in most systems. With the introduction of the Symani^®^ surgical system (Medical Microinstruments, S.p.A, Calci, Pisa, Italy), a microsurgical robotic platform has recently become commercially available to create microanastomoses, such as free flap tissue reconstructions [7–9]. In this context, the experiments presented in this study should demonstrate the feasibility of the robotic Symani^®^ surgical system on cadaveric porcine hearts, describing user experiences and technical description for possible clinical implementation. In this experimental approach, assistive devices are used to perform coronary anastomoses that support the surgeon in a limited space under 3D visualization [5, 6, 10].

## Methods

**Fig. 1:**
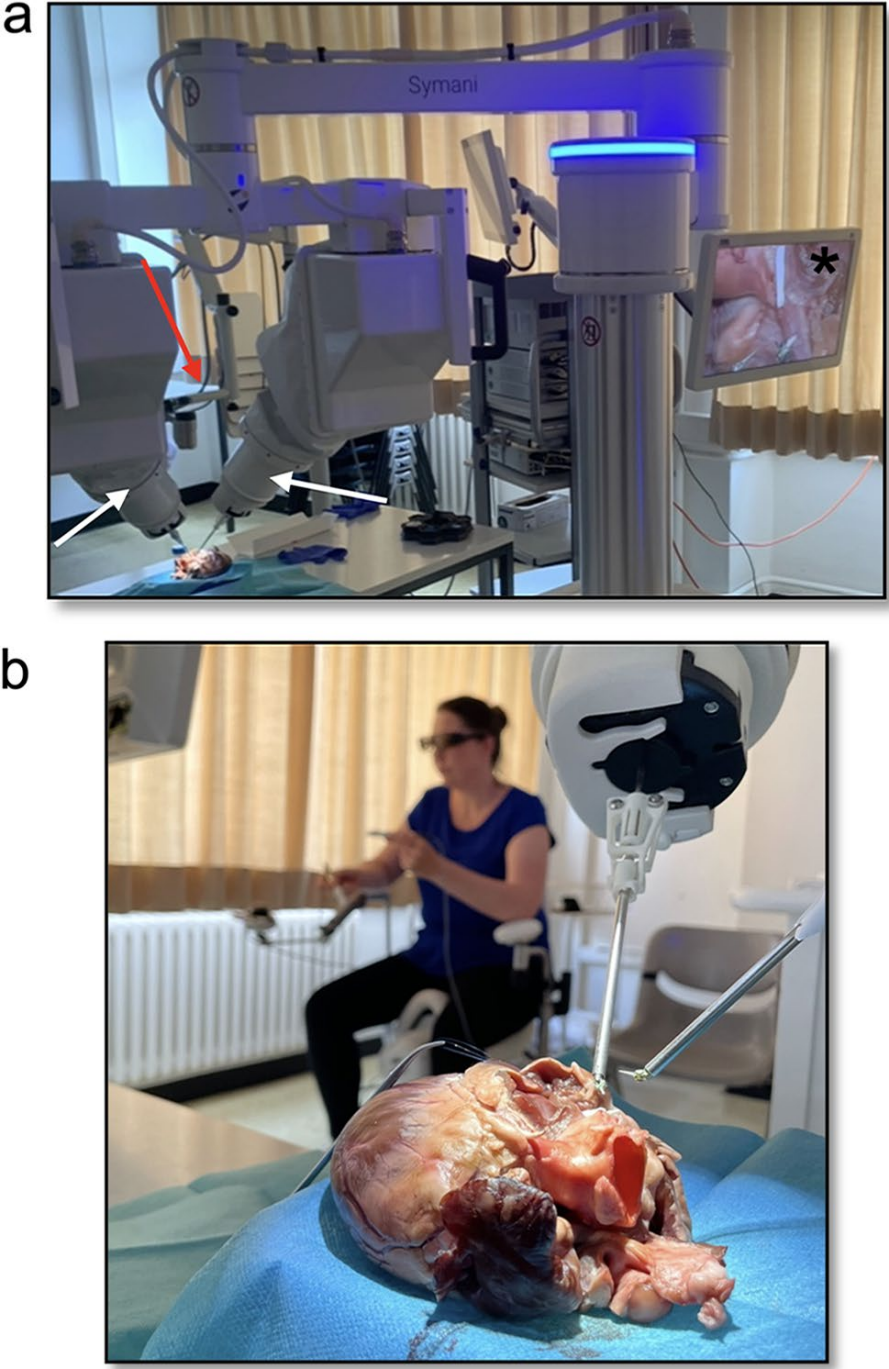
Illustration of the experimental setup of the Symani^®^ robotic system. **a** Positioning of the robotic arms (white arrows), the optical system (red arrow) and the monitor system for 3D visualization (asterisk). **b** Positioning of the surgeon with the exoscopic 3D visualization system in the distance to the surgical setup

**Fig. 2:**
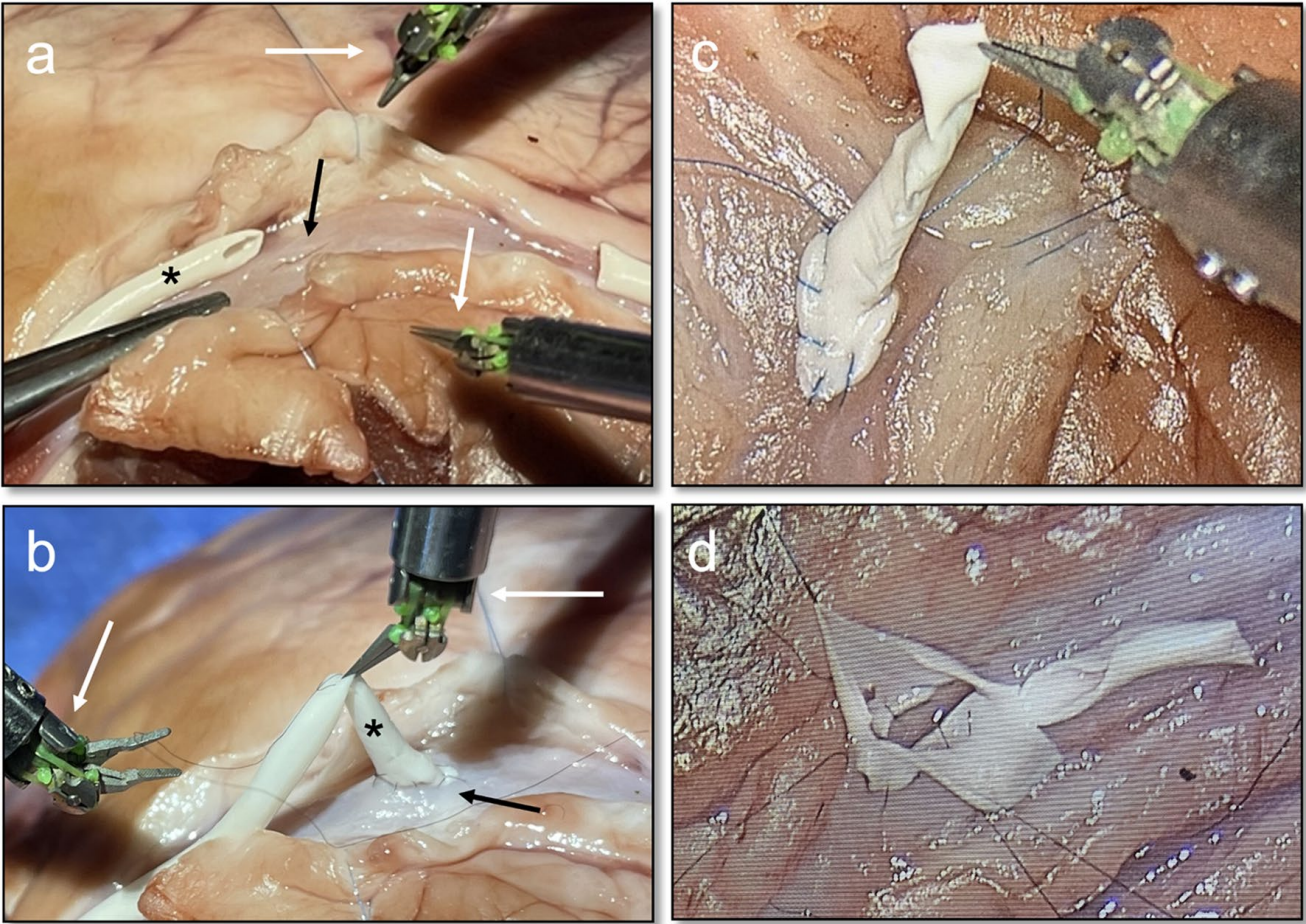
**a**/**b** Preparation of the bypass anastomosis (asterisk) for the vascular incision of the coronary artery (dark arrow). Illustration of the Symani^®^ robotic system and micromanipulators holding the NanoWrist^®^ instruments (white arrows). **c** Completion of the anastomosis using 3D endoscopic visualization. **d** Reopening the bypass to inspect the anastomosis region

The study was approved by the local ethics committee of the University Medical Center Schleswig–Holstein, Kiel, Germany (protocol identification: D 642/23). All surgical procedures were performed on three porcine hearts obtained from an institutional experimental animal facility. Previously, the hearts were prepared on a separate table to allow access to the left main coronary artery over a total distance of 6 cm. The Symani^®^ surgical system (Medical Microinstruments, S.p.A, Calci, Pisa, Italy) with the micromanipulators holding the NanoWrist^®^ instruments was placed in front of the porcine hearts after preparation, so that both robotic arms had free access to the coronary vessels (Fig. 1a). The exoscope was also placed centrally over the anastomosis area. The image section was transferred to a separate screen and converted into a 3D view, which the surgeon could view with specially designed glasses during anastomosis (Fig. 1b). Before performing the anastomosis, an incision of approximately 2 mm was made using a stab scalpel and scissors (Fig. 2a). Two holding sutures were placed in the pericardial fat tissue for better visualization. The anastomoses were performed with a 1 mm artificial prosthesis (WetLab Inc., Otsu-city Shiga, Japan) using a continuous suture technique (Fig. 2b).

A total of 12 anastomoses were performed by three senior cardiovascular surgeons (four anastomoses per surgeon) with the same level of training using the Symani^®^ surgical system. All anastomoses were performed with 10/0 Prolene sutures (Ethicon, Johnson & Johnson Medical GmbH, Norderstedt, Germany) (Fig. 2c). The anastomosis times of each surgeon were documented and the surgical performance evaluated. After completion of the anastomosis, the bypasses were cannulated and visually checked for leakage by flushing with NaCl.

Finally, the anastomoses were opened again with scissors to inspect the resulting tissue quality (Fig. 2d). Success of the procedure was defined by completion of the coronary anastomosis and impartibility (tested by NaCl injection) of the anastomosis. Evaluation of the completed anastomosis was assessed by applying the slightly modified northwestern objective microanastomosis assessment tool (NOMAT) focusing only on the sufficiency and quality of anastomosis (Items XII–XIV, range 0–15), subjective (range 0–100) and in vivo viability grading (pass/fail) made by two independent surgical trained observers via video of the procedures [11].

## Results

**Fig. 3:**
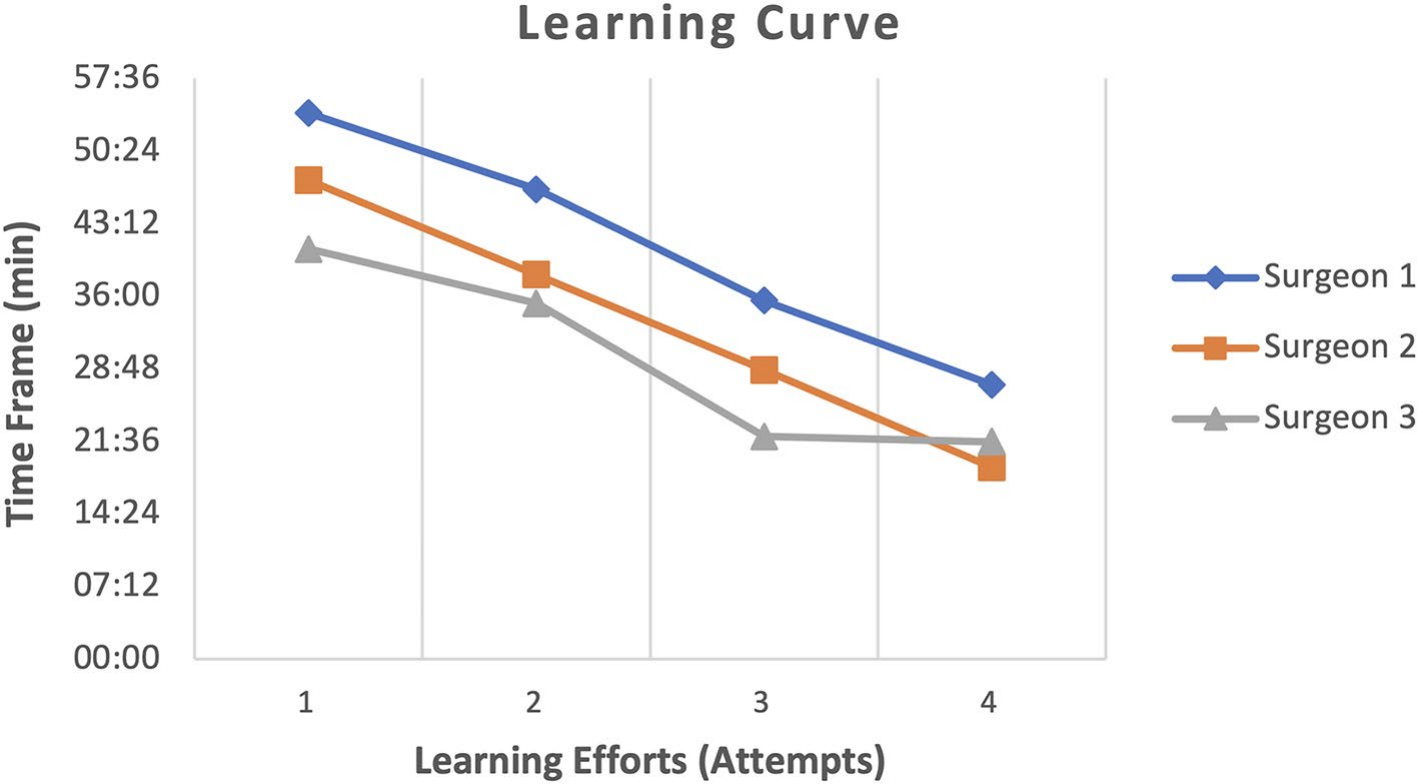
Illustration of the learning curve for the different surgeons during their 4 attempts

The anastomoses were performed on the coronary arteries without technical failures. Placement of the micromanipulators holding the NanoWrist^®^ instruments in combination with the 3D camera was straightforward and could be established tensely at each anastomosis. All three surgeons showed a significant improvement in anastomosis time during the experimental series. The median anastomosis time at the beginning was 47:28 ± 5:30 min and could be reduced to 22:37 ± 3:25 min over the further trial period (Table 1). The improved handling of the needle using 3D visualization had a positive effect on the learning curve in the later attempts (Fig. 3). The longest anastomosis time was 54:11 min at the first attempt and the fastest anastomosis time was 19:02 min at the last attempt (different surgeons).

**Table 1:** Duration of the anastomosis time per attempt with the overall tightness result

	Attempt 1	Attempt 2	Attempt 3	Attempt 4	Leckage test (%)
Surgeon 1	54:11	46:38	35:35	27:14	75
Surgeon 2	47:31	38:09	28:41	19:02	75
Surgeon 3	40:42	35:18	22:06	21:36	100

Attempt in min: sec

At this point, the experience with the system was already slightly advanced, so that an improvement of the time axis due to the greater experience in handling the system is also reflected here. Leak testing of all performed anastomoses also showed an improvement in quality with the increasing experience of the surgeon. Testing the tightness of the anastomoses demonstrated leakage in only two operators in the first attempt. Sufficient anastomoses were verified in the leakage test in the following test series (Table 2). Evaluation of the Symani® surgical system showed considerable advantages in the assessment of stitch distances, particularly in the 3D display. This allowed us to reduce stitch distances to a maximum of 6 stitches depending on the diameter of the opening area with a positive leakage test.

**Table 2:** Leakage test after completion of the anastomosis for each attempt

	*Leckage test			
	Attempt 1	Attempt 2	Attempt 3	Attempt 4
Surgeon 1	neg	pos	pos	pos
Surgeon 2	neg	pos	pos	pos
Surgeon 3	pos	pos	pos	pos

*Leckage test: neg: anastomosis leaking, pos: anastomosis tight

Grading of the performed surgery via the northwestern objective microanastomosis assessment tool (NOMAT) for surgical handling and sufficiency showed rates in the upper range with good user feedback (Table 3). Accordingly, inspection of the anastomosis after surgery showed no signs of high-grade stenosis or leakages (Fig. 2d).

**Table 3:** Northwestern Objective Microanastomosis Assessment Tool (NOMAT) for surgical handling and sufficiency of the vascular anastomosis

	MMI Surgery
Mean NOMAT (range)	11.2 (8–14)
Mean subjective grade (range)	71.2 (53–74)
In vivo viability	10 pass, 2 fail
Mean anastomosis time	34:44 ± 04:35

*NOMAT* northwestern objective microanastomosis assessment tool, Mean anastomosis time in min: sec

## Discussion

The use of minimally invasive robotic surgery compared to open surgery still represents a major challenge for surgeons in the vascular field [12, 13]. Other surgical disciplines, particularly general surgery, urology and gynecology, have already established minimally invasive robotic systems as the gold standard in a bride range of procedures [1]. The Symani^®^ surgical system used in this study has already been successfully used for free flap surgery in oral and maxillofacial surgery and has shown satisfactory results to date [14, 15].

In the cardiovascular field, the Symani^®^ surgical system can certainly be used to perform microanastomoses with previous preparation of the situs in special indications. Particularly in the case of small caliber anastomoses in coronary surgery as well as in shunt surgery and crural surgery, a field of application could develop which should be considered more closely in future. Significant technical advantages of robot-assisted 3D methods compared to open surgery are the high-precision movements of the manipulators and the 3D visualization with multiple magnifications, which enables a vascular anastomosis with sufficient speed in a limited area of the human body [3, 16, 17]. In the final evaluation of the system in this study, the handling of the robotic arms was rated as very direct and focused. Furthermore, the 3D imaging showed a better perception of space and the surgeon was able to manage and implement both the distances and the handling of the tissue more effectively. The optimized visualization of the tissue and the precise sequence of movements made it possible to successfully create sufficient anastomoses with an increasing learning curve. Compared with other robotic-assisted systems in the cardiovascular field, similar anastomosis times are shown to be associated with operator experience [18]. The reported learning curves regarding preparation and use of the systems are also similar [19, 20]. The reduction of leaks in the anastomoses was also reduced as the learning curve increased. However, it should be noted that this system is designed for microanastomoses and therefore the range of application was designed for a very limited radius.

In summary, the system was rated as very intuitive and very suitable for creating anastomoses with good 3D reflection. 3D visualization in particular expands the surgical perspective and leads to more precise and gentle handling of the tissue. The Symani^®^ surgical system is currently being further developed to include new indications in different specialist areas.

Nevertheless, there is still a lack of randomized studies comparing open with minimally invasive endoscopic robot-assisted surgery, and corresponding data for cardiovascular surgery are rare or unavailable [12, 21]. The use of the Symani^®^ surgical system in the area of microanastomoses certainly represents a possibility in coronary surgery as well as in other fields of cardiac surgery (CABG, MVR, tumor resection and ASD repair) [21]. Limited by the surgical radius of the instruments, further studies are required to define the application field. In the field of vascular surgery, operations on structures close to the surface, such as the creation of AV fistulas in the forearm area or for distal crural anastomoses, is conceivable. 3D visualization in particular offers a possible expansion of treatment options due to the improved perspective and more precise visualization. However, the limited application range of the instruments in the operating field should be considered, which restricts the use of the system. Nevertheless, this system shows great potential in the application of microanastomoses in the cardiovascular field and should be explored in further experimental studies.

## Conclusion

Until now, robotic-assisted procedures have not been able to establish themselves as a standard procedure in cardiovascular surgery. In future, 3D visualization of the Symani^®^ surgical system could significantly support surgeons in the lack of haptic feedback, especially in microsurgery. The continuous development of robotic-assisted techniques could lead to new indications in the cardiovascular field in specialized centers, especially with the improved visualization of microanastomoses. In this context, the use of controlled randomized studies is essential. 
